# MEKK2 and MEKK3 suppress Hedgehog pathway-dependent medulloblastoma by inhibiting GLI1 function

**DOI:** 10.1038/s41388-018-0249-5

**Published:** 2018-04-17

**Authors:** Jinqiu Lu, Liansheng Liu, Mingjie Zheng, Xiaoling Li, Ailing Wu, Qingzhe Wu, Cheng Liao, Jian Zou, Hai Song

**Affiliations:** 1 Life Sciences Institute and Innovation Center for Cell Signaling Network, Zhejiang University, Hangzhou, 310058 China; 2 Eye Center of the Second Affiliated Hospital School of Medicine, Institutes of Translational Medicine, Zhejiang University, Hangzhou, 310058 China; 3 Lung Cancer Translational Research, Discovery Center, Janssen (China) Research & Development Center, A Division of Johnson & Johnson (China) Investment Ltd. 5F, Building 1 (North), Jinchuang Mansion 4560 Jinke, Pudong District, Shanghai, 201210 China

**Keywords:** Cell signalling, Oncogenes

## Abstract

Hedgehog (Hh) pathway plays a pivotal role in diverse aspects of development and postnatal physiology. Perturbation of Hh signaling and activation of GLI1 (glioma-associated oncogene 1), a dedicated transcription factor for Hh pathway, are highly associated with several cancers, such as medulloblastoma and basal cell carcinoma. Dynamic and precise control of GLI1 activity is thus important to ensure proper homeostasis and tumorigenesis. Here we show that MEKK2 (MAP3K2) and MEKK3 (MAP3K3) inhibit GLI1 transcriptional activity and oncogenic function through phosphorylation on multiple Ser/Thr sites of GLI1, which reduces GLI1 protein stability, DNA-binding ability, and increases the association of GLI1 with SUFU. Interestingly, MEKK2 and MEKK3 are responsible for FGF2-mediated inhibition on Hh signaling. Moreover, expression of MEKK2 and MEKK3 inhibits medulloblastoma cell proliferation and negatively correlates with Hh pathway activity in medulloblastoma clinical samples. Together, these findings reveal a novel noncanonical GLI1 regulation and provide a potential therapeutic target for the treatment of cancers with aberrant Hh pathway activation, such as medulloblastoma.

## Introduction

Hedgehog (Hh) signaling is highly conserved from *Drosophila* to mammals and plays a crucial role in many aspects of embryonic development, such as neural tube and limb patterning in vertebrates [1–3]. In postnatal physiology, Hh pathway has key roles in tissue homeostasis and regeneration in the epithelia of the skin, intestine, lung, etc. [4]. Aberrant activation of Hh pathway is associated with various types of human cancer, including basal cell carcinoma, medulloblastoma, etc. [5, 6]. Despite the relevance of these insights to development and disease, substantial gaps still remain in our knowledge of the mechanisms that regulate the response to Hh signaling and crosstalk with other pathways. Elucidating the molecular mechanisms of Hh signaling is essential to advance our fundamental understanding of developmental processes and disease mechanisms.

Hh signaling transduction is initiated through ligand binding and inactivating the Hh receptor PTCH1. This relieves PTCH1 repression on the seven-pass transmembrane protein Smoothened (SMO) and enables SMO to translocate to the cilium tip, driving a signaling cascade that culminates in the production of glioma-associated oncogene (GLI) activators. There are three GLI zinc finger transcription factors mediating Hh responses downstream from SMO in mammals [7]. Suppressor of fused (SUFU) is a major negative regulator of Hh singling through controlling GLI protein level and activity [3]. GLI3 repressor generated by proteolysis silences Hh target gene expression in the absence of Hh signaling [8]. Hh signaling inhibits the production of GLI repressor and also facilitates the generation of GLI activitors (mainly from GLI2) to activate Hh target genes, including *GLI1*, *PTCH1*, and *HHIP*, which also contribute to a feedback loop that regulates Hh signaling [2, 7]. GLI1 only functions as transcriptional activator. GLI2 and full-length GLI3 can function as an activator, but the contribution of GLI3 activator to the activity of Hh pathway is insignificant in vivo [9].

Misregulation of GLI leads to developmental and pathological defects, including several cancers. A great progress has been made in the development of SMO antagonists for treating Hh-dependent tumors. However, they have been shown to have several limitations due to the activation of downstream SMO pathway or the occurrence of drug-resistant SMO mutations. Recently, targeting GLI proteins has elicited particular interest to overcome anti-SMO resistance [10]. Although Hh pathway is central to the transcriptional control of GLI target genes, increasing evidence suggests that the posttranslational modification of GLI proteins and noncanonical mechanisms that are apparently independent of Hh signaling also regulate GLI activity [11]. This Hh-independent regulation of GLI function might represent crosstalk between Hh pathway and other signalings during development and tumorigenesis, and could offer opportunities to develop novel targeted therapy for disease conditions with aberrant GLI activity. Further characterization of the Hh–PTCH1–SMO axis-independent regulation of GLI proteins coupled with functional studies is required to advance our mechanistic understanding of Hh signaling and regulations on GLI proteins.

MEKK2 and MEKK3 are two highly homologous mitogen-activated protein kinase kinase kinase (MAP3Ks) among 19 MAP3K super family in the mammalian genome [12]. MEKK3 is 95% conserved in its kinase domain and 55% conserved in overall amino-acid sequence to MEKK2. MEKK2 and MEKK3 are the only MAP3Ks that encode PB1 (an evolutionarily conserved protein–protein interaction motif) domain [12]. MEKK2 and MEKK3 are co-expressed in many cell types, and share substrate specificity.

Here we presented evidence that MEKK2/3 play a critical role in regulating Hh pathway by interacting specifically with GLI1 and phosphorylating it directly. Phosphorylation of GLI1 by MEKK2/3 results in ablation of GLI1 transcriptional activity. Moreover, we found that MEKK2/3 mediate the crosstalk between Hh and fibroblast growth factor (FGF) signalings. Finally, our study revealed that MEKK2/3 suppress Hh signaling-dependent medulloblastoma tumor cell growth. The findings decipher the importance of the posttranscriptional regulation of GLI1, and advance our understanding of the signal transduction mechanism of Hh pathway and Hh pathway activation-related cancers.

## Results

### MEKK2/3 specifically inhibit GLI1 transcriptional activity through their kinase activity

**Fig. 1:**
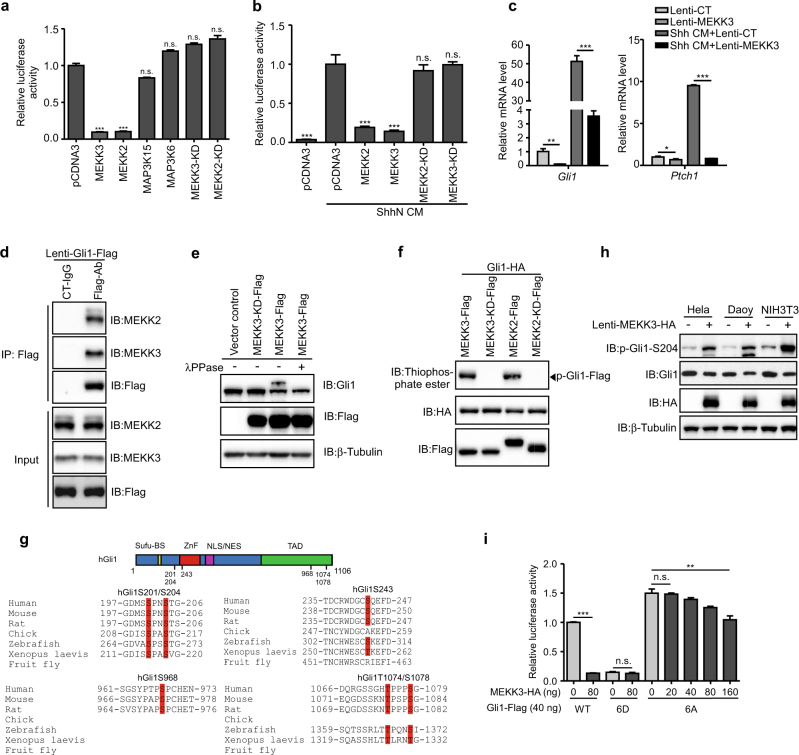
MEKK2/3 inhibit GLI1 transcriptional activity through their kinase activity. **a** MEKK2 and MEKK3 inhibited GLI1 transcriptional activity in HEK293T cells transfected with GliBS-luc reporter, GLI1, and indicated plasmids by a luciferase assay. **b** MEKK2 and MEKK3 inhibited Shh signaling activity in NIH3T3 cells transfected with GliBS-luc reporter and indicated plasmids by a luciferase assay. **c** MEKK3 inhibited expression of Hh signaling target genes *Gli1* and *Ptch1* in NIH3T3 cells transduced with MEKK3 lentivirus assayed by qRT-PCR analysis. **d** Endogenous MEKK2 and MEKK3 proteins were immunoprecipitated by GLI1-Flag proteins. Lysates from GLI1-Flag stable NIH3T3 cells were immunoprecipitated and immunoblotted as indicated. **e** Overexpression of MEKK3 induced a mobility shift of endogenous GLI1 in NIH3T3. Six percent SDS-PAGE was used to examine the mobility shift of GLI1. **f** MEKK2 and MEKK3 promoted phosphorylation of GLI1 in an in vitro kinase assay. MEKK2-Flag, MEKK3-Flag, and GLI1-HA proteins were synthesized using rabbit reticulocyte lysate system in vitro. Total phosphorylation of GLI1 was detected by immunoblotting with thiophosphate ester antibody, which identifies the alkylated thiophosphorylation on GLI1. **g** Alignment of identified phosphorylation sites in GLI1 by mass spectrometry across different species. Blanks indicate that there is no homolog sequence. Schematic representation of GLI1 molecule and phosphorylation sites (upper panel). Some GLI1 structural motifs, including SUFU binding site (SUFU-BS), zinc finger (ZnF), nuclear localization signal (NLS), nuclear export signal (NES), and transcriptional-activation domain (TAD), are denoted. **h** Expression of MEKK3 induced endogenous GLI1 phosphorylation in Hela, Daoy, and NIH3T3 cells. Cell lysates from lentiviral expression of MEKK3 were analyzed by western blot with indicated antibodies. **i** HEK293T cells transfected with GliBS-luc reporter and indicated plasmids were analyzed using a luciferase assay to measure GLI1-6A and GLI1-6D transcriptional activity. *P < 0.05, **P < 0.01, and ***P < 0.001 (two-tailed Student’s *t*-test). All data were mean ± s.d. from representative of three independent experiments conducted in triplicate

To identify kinases that may participate in the regulation of GLI1 transcriptional activity, we employed a GLI-dependent luciferase reporter system (refer as GliBS-Luc) containing eight repeats of GLI protein DNA-binding sequences through which the production of the luciferase reporter is regulated [13, 14], to evaluate the influence of kinases on the GLI1 transcriptional activity. Using this assay, 405 human kinases were individually co-expressed with GLI1 and GliBS-Luc reporter in HEK293T cells (Supplementary table1). In this screening, we found that MEKK2 and MEKK3 displayed the most significant suppressive activity on GliBS-Luc reporter. To further confirm the possible involvement of MEKK2 and MEKK3 in GLI1-regulated gene transcription, we performed several assays to examine the effects of MEKK2/3 overexpression on the GliBS-Luc reporter and Hh signaling activity. First, we found that MEKK3 suppressed GliBS-Luc reporter in a dose-dependent manner (Supplementary Figure S1a). Moreover, expression of MEKK2/3, but not other family members, such as MAP3K15 and MAP3K6, inhibited GLI1-dependent gene transcription in GliBS-Luc reporter assay (Fig. 1a). To determine whether inactivation of GLI1-dependent gene transcription by MEKK2/3 depends on their kinase activity, we generated a kinase-dead mutant, MEKK3-KD, which contains S526/T530 to Ala point mutations [15]. Unlike wild-type (WT) MEKK3, the kinase-deficient or deletion of kinase domain mutants did not show any suppressive effect on GLI1 (Fig. 1a and Supplementary Figure S1b). The involvement of MEKK2/3 in gene transcriptional regulation of GLI1 appears to be specific because expression of MEKK2/3 did not suppress GLI2- and GLI3-induced reporter activity (Supplementary Figure S1c).

We also investigated whether MEKK2/3 could inhibit other stimuli-induced GLI1 transcriptional activity. DYRK1A phosphorylates GLI1 and promotes its nuclear localization and transcriptional activity [16]. We showed that DYRK1A-mediated activation of GliBS-luc activity was dramatically inhibited by MEKK3 expression in HEK293T cells (Supplementary Figure S1d). Importantly, expression of MEKK3 severely inhibited Shh-induced GliBS reporter activity and downstream target gene expression in Hh-responsive NIH3T3 cells (Fig. 1b, c). Because *gli1* rather than *gli2* is the principal activator of Hh signaling in early zebrafish embryos [17], we used the zebrafish system as a readout to assess the in vivo function of *mekk2/3*. We found that MEKK3 overexpression resulted in reduced *ptch1* expression in brain area and loss of *ptch1* expression in fin buds (Supplementary Figure S1e), in which Hh signaling perturbation leads to well-characterized phenotype [18, 19]. Furthermore, injection of gRNAs for *mekk2* and *mekk3* with *Cas9* mRNA into zebrafish single-cell embryos led to knockout of *mekk2* and *mekk3* (Supplementary Figure S1f). In the *mekk2/3* double-knockout (DKO) zebrafish embryos, there was an elevation of *gli1* and *hip1* mRNA levels compared to the control group (Supplementary Figure S1g). These results suggest a negative role of MEKK2/3 in controlling Hh signaling through their kinase activity.

### MEKK2/3 associate with GLI1 and phosphorylate it at multiple sites

To further investigate the molecular mechanisms how MEKK2/3 regulate GLI1 activity, we tested a model whether MEKK2/3 associate with GLI1 and phosphorylate it. Indeed, we found that GLI1 interacted with MEKK2 and MEKK3 in a co-immunoprecipitation assay (Supplementary Figures S1h-S1l). Furthermore, GLI1-Flag proteins efficiently immunoprecipitated endogenous MEKK2 and MEKK3 in GLI1-Flag stable NIH3T3 cells generated by lentiviral transduction (Fig. 1d). Notably, expression of MEKK3, but not kinase-deficient MEKK3, induced a mobility shift of GLI1 in SDS-polyacrylamide gel electrophoresis (SDS-PAGE; Fig. 1e and Supplementary Figure S1m), indicating MEKK3 might phosphorylate GLI1. Because MEKK2/3 are serine/threonine kinases and their kinase activity is essential for GLI1 transcriptional activity, we wanted to know whether MEKK2/3 could phosphorylate GLI1 protein. To test this, we performed an in vitro kinase assay. As expected, significant phosphorylation of GLI1-Flag was observed in the presence of MEKK2 or MEKK3 (Fig. 1f), indicating that MEKK2 and MEKK3 directly phosphorylate GLI1.

To identify which amino acids in GLI1 were phosphorylated by MEKK2/3, we performed mass spectrometric analysis using GLI1-Flag proteins purified from cells co-expressing GLI1 with either MEKK3-WT or MEKK3-KD. Mass spectrometry showed that GLI1 was phosphorylated at sites Ser201, Ser204, Ser243, Ser968, Thr1074, and Ser1078 by MEKK3-WT but not the KD mutant (Supplementary Figure S1n). Notably, except in *Drosophila*, almost all the phosphorylated sites are conserved across different species (Fig. 1g). Interestingly, MEKK2/3 are not found in *Drosophila*. It is thus possible that MEKK2/3 represent vertebrate-specific regulators of GLI1 and reflect pathway divergence. To further investigate endogenous GLI1 phosphorylation by MEKK2/3, we developed a rabbit polyclonal antibody that specifically recognizes phosphorylated Ser204 of GLI1 (p-GLI1-S204). This antibody recognized WT GLI1-Flag but not GLI1-S204A-Flag co-expressed with MEKK3 (Supplementary Figure S1o), indicating that this antibody is highly specific. Using this antibody, we found that overexpression of MEKK3 effectively induced Ser204 phosphorylation of endogenous GLI1 in Hela, Daoy, and NIH3T3 cells (Fig. 1h).

### Mutating the phosphorylation sites of GLI1 affects GLI1’s transcriptional activity

To examine the function of these sites, we generated serial mutations for GLI1 by changing Ser/Thr to Ala or Asp. We found that only when all six Ser/Thr residues were mutated to Ala (refer as GLI1-6A), phosphorylation of GLI1 by MEKK3 disappeared in an in vitro kinase assay (Supplementary Figure S1p), suggesting that all these sites in GLI1 are phosphorylated by MEKK2/3. Next, we sought to determine the function of these sites on GLI1 transcriptional activity. We found that only GLI1-6A was refractory to MEKK3’s inhibition in the GliBS reporter assay (Fig. 1i and Supplementary Figure S1q). Furthermore, we showed that strikingly, phosphomimetic mutation of GLI1 (refer as GLI1-6D) resulted in a great loss of transcriptional activity in GliBS-luc reporter assay to a level comparable with GLI1-WT co-expressed with MEKK3, whereas various combinations of “1D, 2D, or 3D” mutants moderately reduced it (Fig. 1i and Supplementary Figure S1r). These data indicate that all the phosphorylation sites contribute to the inhibitory effect of GLI1 by MEKK2/3 to a certain extent. These data, coupled with observations from the in vitro kinase assay, proposed a direct MEKK2/3-induced modification on GLI1 at multiple serine and threonine residues, thereby inhibiting its transcription function.

### Deletion of MEKK2/3 leads to the accumulation of GLI1, while overexpression of MEKK2/3 destabilizes GLI1

**Fig. 2:**
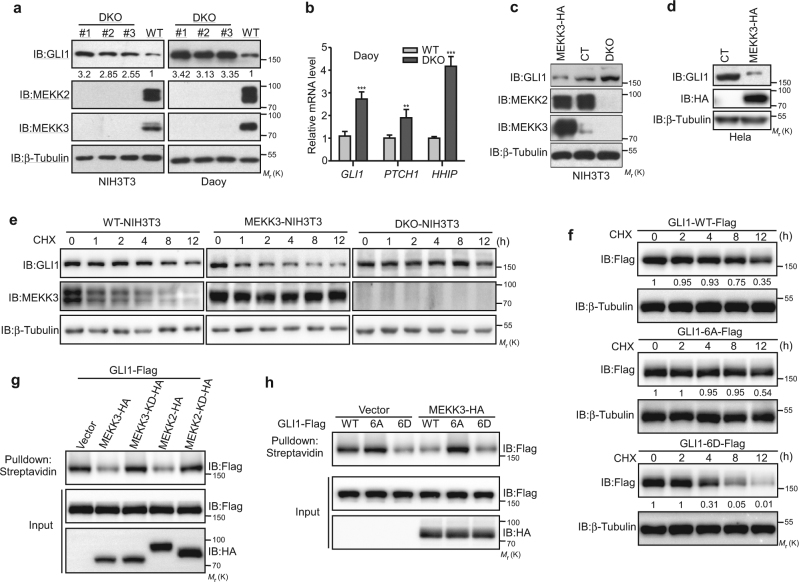
MEKK2/3 destabilize GLI1 protein and inhibit GLI1 DNA-binding ability. **a** GLI1 protein levels were upregulated in NIH3T3 and Daoy DKO cells by western blot analysis with indicated antibodies. **b** *GLI1*, *PTCH1*, and *HHIP* mRNA levels were upregulated in Daoy DKO cells by qRT-PCR analysis. **c**, **d** Overexpression of MEKK3 reduced GLI1 protein levels in NIH3T3 (**c**) and Hela (**d**) cells by western blot analysis with indicated antibodies. **e** NIH3T3 cells were treated with cycloheximide (CHX, 20 μg/ml) for the indicated times, and cell lysates were analyzed by western blot with indicated antibodies. **f** HEK293T cells were transfected with indicated plasmids and then treated with CHX (20 μg/ml) for the indicated times, and cell lysates were analyzed by western blot with indicated antibodies. **g** Expression of MEKK3 and MEKK2 reduced DNA binding of GLI1 in a biotin-labeled DNA pull-down assay. **h** Biotin-labeled DNA pull-down assay for the binding ability of GLI1, Gli1-6A, and GLI1-6D in the presence of MEKK3

Given the profound effect of MEKK2/3-mediated GLI1 phosphorylation, we attempted to understand the underlying mechanism for phosphorylation-mediated GLI1 inhibition. To promote a mechanistic understanding of MEKK2/3 function, we created *MEKK2* and *MEKK3* DKO cell lines in NIH3T3 and Daoy cells using CRISPR/Cas9 system to investigate their contributions to GLI1 activity. Intriguingly, deleting *MEKK2/3* resulted in a significant increase of GLI1 protein levels in both Daoy and NIH3T3 cells (Fig. 2a). Moreover, deletion of *MEKK2/3* resulted in increased *GLI1* and *PTCH1* mRNA levels in Daoy and NIH3T3 cells (Fig. 2b and Supplementary Figure S2a). Conversely, overexpression of MEKK3 greatly reduced endogenous GLI1 protein levels in both NIH3T3 and Hela cells (Fig. 2c, d), and exogenous GLI1 in HEK293T cells (Figure S2b).

Next, we focused on GLI1 protein stability. When MEKK2/3 were intact in NIH3T3 cells, the amount of endogenous GLI1 decreased with a half-life of 8 h (Fig. 2e, left). In cells lacking both MEKK2 and MEKK3, GLI1 remained stable with a half-life of over 12 h (Fig. 2e, right). Strikingly, expression of MEKK3 greatly shortened the half-life of GLI1 to 1 h (Fig. 2e, middle). Moreover, treatment with proteasome inhibitor (MG132) restored the endogenous GLI1 protein levels in MEKK3 overexpression NIH3T3 cells (Supplementary Figure S2c). To further understand how MEKK2/3 control GLI1 activity, we asked whether the phosphorylation status of GLI1 affects its protein stability. Data showed that GLI1-6A was considerably stable, while GLI1-6D, phosphomimetic mutant, was highly unstable in HEK293T cells (Fig. 2f and Supplementary Figure S2d). Further analysis revealed that both the N-(S201/204/243)- and C-(S968/T1074/S1078)-terminal phosphorylation sites are required for the stability of GLI1 (Figure S2e). Therefore, phosphorylation of GLI1 by MEKK2/3 results in destabilization of GLI1.

### MEKK2/3 inhibit GLI1 transcriptional activity partially through reducing GLI1’s DNA-binding ability

Since the Ser243 residue of GLI1 is located in the DNA-binding domain (DBD) and Ser201/Ser204 residues are close to DBD, we were wondering whether MEKK2/3-mediated GLI1 phosphorylation affects GLI1 binding with DNA. By using an in vitro DNA pull-down assay with the GliBS sequence, we indeed observed that the presence of MEKK2 or MEKK3 but not their kinase-dead mutants severely inhibited the binding of GLI1 with GliBS oligonucleotides (Fig. 2g, Supplementary Figure S2f). Furthermore, GLI1-6A mutant was resistant to the inhibitory effect of MEKK3 on GLI1 DNA binding (Fig. 2h). Intriguingly, the DNA-binding capability of GLI1 was severely diminished when GLI1-6D mutant was used to mimic its phosphorylation (Fig. 2h). Furthermore, as shown in Supplementary Figures S2g and S2h, GLI1-S201/S204/S243A and GLI1-S201/S204/S243D displayed similar activity as GLI1-6A and GLI1-6D mutants, respectively. Therefore, our observations support the negative regulation of MEKK3 on GLI1 DNA binding through phosphorylation. This finding suggests that the reduced GLI1 transcriptional activity is partially due to its inefficient binding to Hh-responsive promoters.

### Phosphorylation of GLI1 by MEKK2/3 leads to cytoplasmic retention of GLI1

**Fig. 3:**
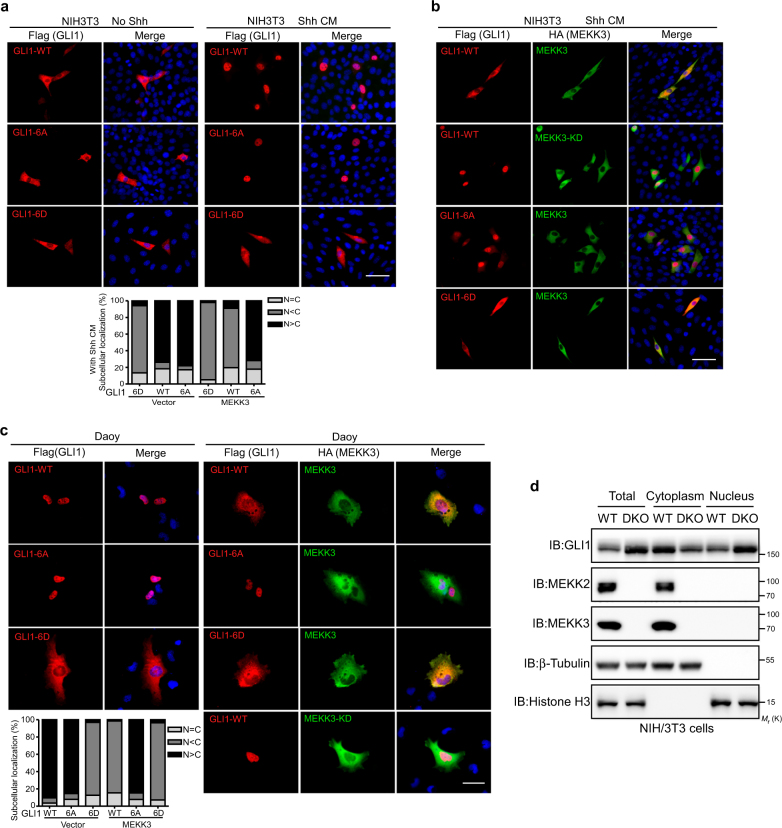
MEKK2/3 retain GLI1 in cytoplasm through phosphorylation of GLI1. **a** Immunofluorescent analysis of the localization of GLI1 and its mutants in NIH3T3 cells with or without Shh CM treatment. Quantification of immunofluorescent analysis was shown in lower panel. **b** Immunofluorescent analysis of the localization of GLI1 and its mutants with MEKK3 expression in NIH3T3 cells treated with Shh CM. **c** Immunofluorescent analysis of the localization of GLI1 and its mutants in Daoy cells with or without MEKK3 expression. Quantification of immunofluorescent analysis was shown in lower panel. **d** Western blot analysis of GLI1 protein levels in the nuclear and cytoplasmic fractions in WT and DKO NIH3T3 cells. Scale bar = 25 μm

GLI proteins display dynamic shuttling between the nucleus and cytoplasm, and execute their transcriptional function in the nucleus. Controlling the localization of GLI is one of the means to regulate Hh signaling activity. We thus performed immunofluorescence assay to examine the effect of MEKK2/3 on subcellular localization of GLI1. As shown in Fig. 3a, resting-state GLI1 was mainly distributed in the cytoplasm in NIH3T3 until the cells were activated by Shh, which induced the nuclear enrichment of GLI1. GLI1-6A exhibited similar response to Shh as GLI1-WT (Fig. 3a). However, GLI1-6D remained in cytoplasm regardless of Shh treatment (Fig. 3a). Strikingly, when co-expressed with MEKK3 or MEKK2, GLI1 did not respond to Shh stimulation and still stayed in cytoplasm (Fig. 3b, Supplementary Figure S3a). Importantly, GLI1-6A responded normally to Shh in the presence of MEKK3, indicating a resistance to MEKK3’s inhibition (Fig. 3b). Since both MEKK3-KD and MEKK3-WT associate with GLI1 (Supplementary Figure S1l), these results indicate that GLI1’s cytoplasm retention is due to MEKK3’s kinase activity, but not sequestering GLI1 in cytoplasm by physical interaction.

To further confirm these observations, we tested the regulation of GLI1 localization by MEKK2/3 in Daoy cells. Unlike in NIH3T3, GLI1 and GLI1-6A were mainly localized in nucleus in Daoy cells without Shh stimulation (Fig. 3c). However, GLI1 but not GLI1-6A translocated to cytoplasm in the presence of MEKK3 in Daoy cells (Fig. 3c). Importantly, GLI1-6D remained in cytoplasm regardless of MEKK3 expression in Daoy cells (Fig. 3c). Nuclear and cytoplasmic fractionation analysis for GLI1 distribution reciprocated the immunofluorescence experiments. In DKO NIH3T3 and Daoy cells, deletion of MEKK2/3 resulted in more GLI1 accumulated in the nucleus (Fig. 3d and Supplementary Figure S3b). Taken together, these data suggest that phosphorylation of GLI1 by MEKK2/3 prevents the GLI1 nuclear accumulation, thus inactivating the expression of its target genes.

### MEKK2/3 enhance the binding between SUFU and GLI1

**Fig. 4:**
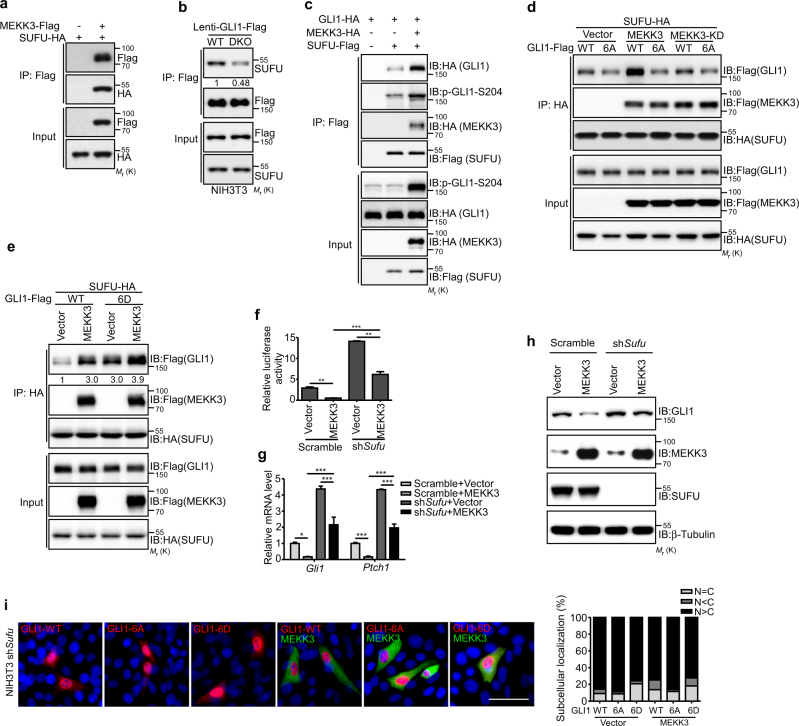
Phosphorylation of GLI1 by MEKK2/3 increases its binding with SUFU. **a** MEKK3 was associated with SUFU in a co-immunoprecipitation assay in HEK293T cells transfected with indicated plasmids. **b** GLI1 bound less to SUFU in DKO NIH3T3 cells. The endogenous SUFU was immunoprecipitated by GLI1-Flag expressed from lentivirus vector in CT and DKO NIH3T3 cells. **c** MEKK3 expression increased binding between GLI1 and SUFU in HEK293T cells in a co-immunoprecipitation assay. **d** GLI1-6A bound less to SUFU in HEK293T cells in a co-immunoprecipitation assay. **e** GLI1-6D displayed stronger SUFU binding than GLI1-WT in HEK293T cells in a co-immunoprecipitation assay. **f** *Sufu* knockdown NIH3T3 cells were transfected with GliBS-Luc and indicated plasmids, and followed by GliBS reporter assay. *Sufu* knockdown led to increased GliBS reporter activity. MEKK3 inhibited GliBS-luc reporter activity in *Sufu* knockdown NIH3T3 cells. **g** *Gli1* and *Ptch1* mRNA levels were analyzed by qRT-PCR from *Sufu* knockdown NIH3T3 cells with overexpression of MEKK3. **h** Western blot analysis of NIH3T3 lysates derived from control and *Sufu* knockdown NIH3T3 cells with overexpression of MEKK3. **i** Immunofluorescent analysis of the localization of GLI1 in *Sufu* knockdown NIH3T3 cells. Quantification of immunofluorescent analysis was shown in right panel. *P < 0.05, **P < 0.01, and ***P < 0.001 (two-tailed Student’s *t*-test). Quantitative data were presented as mean ± s.d. from a representative of at least three independent experiments. Scale bar = 25 μm

SUFU, a major negative regulator of mammalian Hh signaling, has been shown to sequester GLI proteins in the cytoplasm [20–23]. The exclusive cytoplasmic localization of GLI1-6D suggests that SUFU may involve in the GLI1 cytoplasmic retention. To investigate the role of SUFU in MEKK2/3-mediated inhibition, we first tested whether MEKK3 associates with SUFU. The co-immunoprecipitation experiment showed that MEKK3 indeed interacted with SUFU (Fig. 4a). Although MEKK3 associated with SUFU, we were unable to detect substantial kinase activity for SUFU (Supplementary Figure S4a). We therefore investigated whether GLI1 phosphorylation by MEKK2/3 affects its binding with SUFU. First, we found that there was less SUFU associated with GLI1 in DKO NIH3T3 cells (Fig. 4b). Furthermore, the interaction between SUFU and GLI1 was markedly increased in HEK293T cells expressing MEKK3 (Fig. 4c). Similarly, SUFU interacted more strongly with GLI1-6D than GLI1-WT and GLI1-6A (Fig. 4d, e). Importantly, expression of MEKK3 did not enhance GLI1-6A binding with SUFU (Fig. 4d). Moreover, GLI1-S201/204/243A and GLI1-S201/204/243D mutants exhibited similar affinity with SUFU as GLI1-6A and GLI1-6D, respectively (Supplementary Figure S4b and S4c). All these data suggested that the phosphorylation of GLI1 by MEKK2/3 enhances its binding to SUFU.

### MEKK2/3 inhibit GLI1 transcriptional activity even with nuclear localized GLI1 caused by the elimination of SUFU

Hh stimulation releases GLI1 from SUFU and leads to GLI1 nuclear accumulation and expression of target genes. Moreover, loss of SUFU leads to pathway activation-independent Hh stimulation. We then asked whether MEKK2/3 inhibit GLI1 activity in the absence of SUFU. Interestingly, we detected significant suppression of GLI1 transcriptional activity by MEKK3 in *Sufu *knockdown NIH3T3 cells using GliBS-luc reporter assay (Fig. 4f). Moreover, MEKK3 inhibited Hh target gene expression in *Sufu*-deficient NIH3T3 cells (Fig. 4g). Of note, inhibition of GLI1 activity and protein level by MEKK3 in *Sufu* knockdown cells did not reach maximal level of inhibition as in control cells (Fig. 4f–h). Furthermore, we tested whether MEKK2/3-mediated GLI1 cyotoplasmic retention is dependent on SUFU. The results showed that GLI1 was localized in nucleus in *Sufu*-deficient NIH3T3 cells regardless the expression of MEKK3 (Fig. 4i), indicating that SUFU is required for GLI1 cytoplasmic localization regulated by MEKK2/3. Although GLI1 was in nucleus in *Sufu*-deficient NIH3T3 cells, MEKK2/3 still inhibited GliBS reporter activity and target gene expression, indicating that phosphorylated GLI1 by MEKK3 possesses much lower transcriptional activity in nucleus, possibly due to the reduced DNA-binding ability. In conjunction with our observation of GLI1 localization (Fig. 3a–c), these data suggest that SUFU is required for GLI1 cytoplasmic retention regulated by MEKK2/3, and phosphorylation of GLI1 by MEKK2/3 exhibits reduced transcriptional activity in nucleus.

### FGF2 inhibits Hh signaling and proliferation of Daoy cells through activating MEKK2/3

**Fig. 5:**
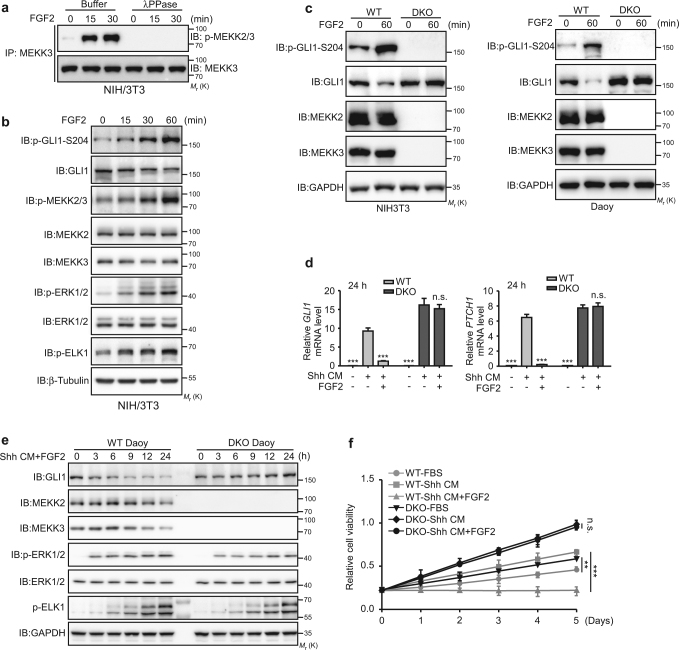
MEKK2 and MEKK3 are required for FGF2-mediated GLI1 transcriptional inhibition. **a** MEKK3 proteins were immunoprecipitated from FGF2-stimulated NIH3T3 cells, treated with λ phosphotase and analyzed with anti p-MEKK2/3 antibody. **b** Western blot analysis of NIH3T3 cells treated with FGF2 (50 ng/ml) by indicated antibodies. **c** NIH3T3 and Daoy cells treated with FGF2 were analyzed with anti p-GLI1-S204 antibody. Of note, deletion of MEKK2/3 completely abolished S204 phosphorylation of GLI1. **d**, **e** WT and DKO Daoy cells were treated with Shh CM and FGF2, and subjected to western blot analysis (**d**) and qRT-PCR analysis (**e**). **f** Cell proliferation analysis of WT Daoy and DKO Daoy cells treated with FGF2 and Shh CM. **P < 0.01, and ***P < 0.001 (two-tailed Student’s *t*-test). Quantitative data were presented as mean ± s.d. from a representative of at least three independent experiments

Next, we asked whether activation of MEKK2 and MEKK3 at physiological condition is able to inhibit Hh pathway activity. FGF2 has been shown to activate MEKK2 in osteoblasts [24]. Interestingly, FGF2 blocks Shh signaling in neuronal precursors and tumor cells [25]. These studies suggest that inhibition of Hh signaling by FGF2 might be through MEKK2 and MEKK3. To test this, we examined whether FGF2 could activate MEKK2 and MEKK3. We showed that FGF2 markedly induced the phosphorylation of MEKK3 after FGF2 treatment using anti-phospho-MEKK2/3 antibody and phos-tag SDS-PAGE assay (Fig. 5a, Supplementary Figure S5a), demonstrating the activation of MEKK3 kinase activity by FGF2 stimulation. Next, we investigated whether FGF2 could inhibit GLI1 activity and Hh signaling. We found that FGF2 promoted GLI1 protein degradation in NIH3T3 and Daoy cells (Fig. 5b, e). Furthermore, priming of Daoy cells with Shh for 9 h induced a significant upregulation of GLI1 target genes at the transcript level, which was gradually reduced after treatment with FGF2 in Daoy cells (Supplementary Figure S5b).

To assess whether FGF2 could induce GLI1 phosphorylation in vivo and the role of MEKK2/3 in this process, we performed western blot analysis with anti p-GLI1-S204 antibody using NIH3T3 and Daoy cells treated with FGF2. The results showed that the phosphorylation of GLI1 at S204 was readily detected in WT NIH3T3 and Daoy cells treated with FGF2 but not in DKO NIH3T3 and Daoy cells (Fig. 5c). Notably, the basal level of GLI1-S204 phosphorylation was completely abolished in DKO NIH3T3 and Daoy cells (Fig. 5c), which implies that MEKK2/3 are absolutely required for GLI1 phosphorylation at S204. To further elucidate the crosstalk between FGF2 and Shh signaling, GLI1 protein levels and its target gene expression were examined in DKO Daoy cells treated with Shh and FGF2 in a time-course experiment. Interestingly, elimination of both MEKK2 and MEKK3 in Daoy cells completely abolished FGF2-mediated inhibition on GLI1 protein and expression of its target genes (Fig. 5d, e). Furthermore, FGF2 inhibited Shh-induced Daoy cell proliferation but not in DKO cells (Fig. 5f). Therefore, our results indicate that MEKK2/3 mediate the crosstalk between Hh and FGF pathways, and FGF2–MEKK2/3–GLI1 signaling axis is independent of canonical Hh signaling in the regulation of GLI1 activity.

### MEKK2/3 inhibit Hh pathway-dependent tumor cell proliferation

**Fig. 6:**
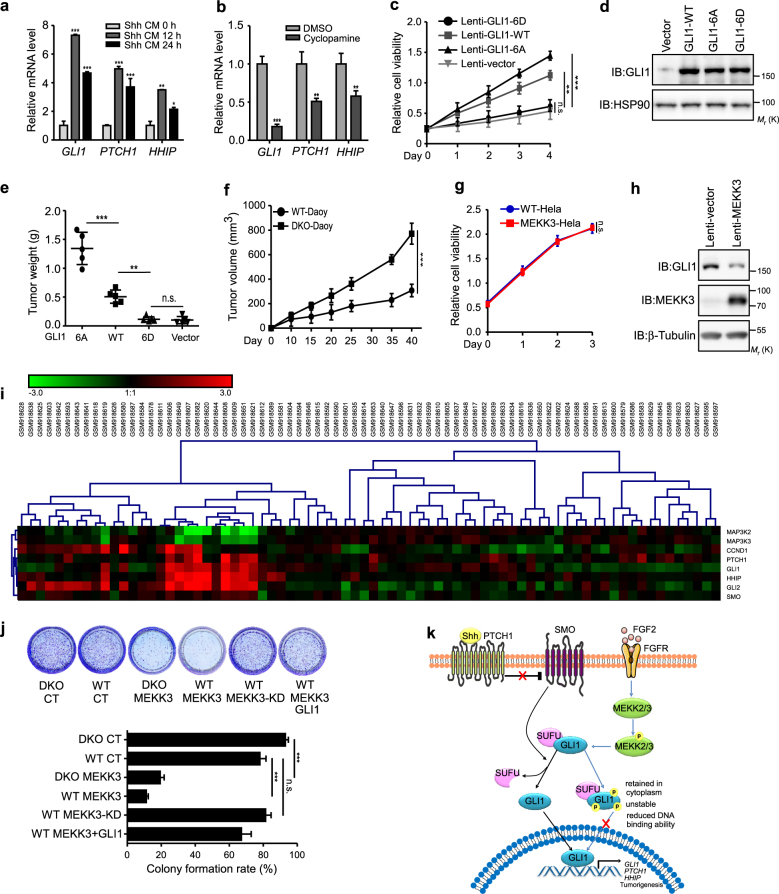
MEKK2/3 inhibit Hh pathway-dependent medulloblastoma tumor cell proliferation. **a** qRT-PCR analysis of Daoy cells treated with Shh CM for indicated times. **b** Daoy cells were treated with cyclopamine (5 μM) for 12 h and subjected for qRT-PCR analysis. **c** Daoy cells stably expressing GLI1-WT, GLI1-6A, and GLI1-6D were subjected to cell proliferation assay using CCK8. **d** Cell lysates from GLI1-WT, GLI1-6A, and GLI1-6D Daoy cells were analyzed by western blot with indicated antibodies. **e** Daoy cells stably expressing GLI1-WT, GLI1-6A, and GLI1-6D were subcutaneously injected into the nude mice (five mice for each group), and tumor weights were measured 20 days later. **f** WT and DKO Daoy cells (5 × 10^6^) were subcutaneously injected into nude mice (six mice for each group), and tumor volume was measured and calculated. **g** Hela cells were transduced with MEKK3 lentivirus and subjected to cell proliferation assay using CCK8. **h** Cell lysates from **g** were analyzed by western blot. **i** Heat map analysis reveals that the expression of MEKK2/3 is negatively correlated with Hh pathway activity in GSE37418 dataset. **j** Daoy cells were transduced with indicated lentivirus and subjected to colony formation assay. **k** The diagram shows that activated MEKK2/3 by FGF2 directly phosphorylate GLI1. Phosphorylation results in reduced GLI1 protein stability, transcriptional activity, and tumorigenesis potency. *P < 0.05, **P < 0.01, and ***P < 0.001 (two-tailed Student’s *t*-test). Quantitative data were presented as mean ± s.d. from a representative of at least three independent experiments

Aberrant activation of Hh signaling has been identified in many human malignancies, including medulloblastoma [2, 7]. Previous studies demonstrate that Daoy cells, a human medulloblastoma cell line, display a full responsiveness to Hh pathway [26]. To further confirm this, we used Shh conditioned medium (Shh CM) or cyclopamine to treat Daoy cells. Consistent with previous study, Daoy cells possess a fully inducible endogenous Hh pathway and present a valuable cancer model for detailed analysis of physiological Hh/GLI1 signaling (Fig. 6a, b and Supplementary Figures S6a, S6b). To investigate the relationship between GLI1 phosphorylation and its tumorigenic functions, we generated GLI1-WT, GLI1-6A, and GLI1-6D stable Daoy cells using lentiviral vector. Consistent with their transcriptional potential, GLI1-6A and GLI1-WT but not GLI1-6D increased cell proliferation in vitro and tumor growth in nude mice (Fig. 6c–e, Supplementary Figure 6c). These results support that the phosphorylation of GLI1 diminishes its function on promoting cell proliferation.

To further investigate the function of GLI1 phosphorylation by MEKK2/3, we examined the effect of MEKK2/3 on Daoy cell proliferation. First, we tested the tumorigenicity of WT and DKO Daoy cells by subcutaneously injecting them into nude mice. Consistent with our in vitro data, deficiency of MEKK2/3 resulted in stronger tumorigenicity of Daoy cells in nude mice (Fig. 6f). Next, we tried to generate MEKK3 stable Daoy cells. However, we only obtained MEKK3-KD stable Daoy clones but not WT MEKK3 (Supplementary Figure S6d), indicating an anti-proliferation activity of MEKK3 in Daoy cells. To circumvent this, we transduced Daoy cells using lentiviral vector to transiently express MEKK3 and performed in vitro analysis using these cells (Supplementary Figures S6e and S6f). In the colony formation and cell proliferation assay, we found that MEKK3 but not MEKK3-KD significantly reduced the colony number and cell proliferation in WT and DKO Daoy cells, and expression of GLI1 partially rescued this inhibition (Fig. 6j and Supplementary Figure S6g). In addition, we found that MEKK3 inhibited cell proliferation in NIH3T3, but not in Hela cells, which were not sensitive to Hh signaling blockade (Supplementary Figures S6h and Fig. 6g, h). Moreover, expression of MEKK3 in another medulloblastoma cell line D341 Med inhibited cell proliferation, expression of Hh pathway target genes, and reduced GLI1 protein levels (Supplementary Figures S6i-l). More importantly, we searched two publicly available medulloblastoma datasets (GSE41842 and GSE37418) and found that there are negative correlations between the expression of MEKK2/3 and Hh pathway target genes in both datasets (Fig. 6i and Supplementary Figure S6m), suggesting the inhibitory role of MEKK2/3 on Hh pathway in clinical patient samples. Taken together, these results indicate that MEKK2/3 inhibit Hh pathway-dependent tumorigenesis through inactivation of GLI1 by phosphorylation.

Collectively, our results suggested that activated MEKK2/3 by FGF2 directly phosphorylate GLI1. Phosphorylation of GLI1 results in reduced GLI1 protein stability, transcriptional activity, and tumorigenesis potency (Fig. 6k).

## Discussion

GLI1 is a key transcriptional target and mediator of Hh signaling. Control of GLI1 expression is critical for cell proliferation and its abnormal regulation leads to tumor progression. Elucidating the molecular mechanism of the regulation of GLI1 is critical for understanding Hh signal transduction, normal embryonic patterning, and pathological conditions such as human congenital anomalies and cancers arising from dysregulated Hh signaling. In this study, we identified a novel regulation of GLI1 by MEKK2/3, in which MEKK2/3 phosphorylate GLI1 at multi-Ser/Thr sites, resulting in its tight association with SUFU and less DNA-binding ability. In addition, our work implies that MEKK2/3 mediate FGF2-induced inhibition on Hh signaling through regulating GLI1 phosphorylation. Since GLI1 is known as an oncogene, our results also provide further evidence to support MEKK2/3 suppress Hh-dependent tumor cell growth by regulating GLI1 activity.

Posttranslational modification of GLI1 contributes to the regulation of GLI1 protein stability, location, transcriptional activity, and association with SUFU. Control of GLI1 protein level and activity provides a major mechanism to modulate Hh signaling. GLI1 has been shown to utilize the Numb-Itch ubiquitination pathway or βTrCP-dependent mechanism for protein degradation [27–29]. Hh signaling leads to SUFU-GLI1 dissociation and GLI1 activation. Our studies demonstrate that MEKK2/3 strengthen GLI1-SUFU interaction, which results in GLI1 cytoplasmic retention (Fig. 4c). MEKK2/3 cannot retain GLI1 in cytoplasm in the absence of SUFU. Indeed, subcellular localization study of GLI1-6D clearly shows that GLI1 is present in nucleus when SUFU is eliminated (Fig. 4i). However, MEKK2/3 still efficiently inhibit GLI1 transcriptional activity in this scenario (Fig. 4f, g), possibly due to the reduced DNA-binding ability of GLI1 by phosphorylation modification. Our studies suggest that MEKK2/3 control GLI1 activity by multiple mechanisms through SUFU-dependent and -independent regulation.

One of the significant findings we have reported is the inhibitory ability of FGF2 on Hh pathway is mediated by MEKK2/3. Previous study showed FGF-mediated inhibition of Shh response requires activation of FGF receptors, and ERK and JNK kinases, because it can be blocked by inhibitors of these enzymes in granule cell precursors [25]. Notably, in our study, FGF2-induced inhibition on GLI1 activity and cell proliferation is dampened in DKO Daoy cells even with the intact RAS/MEK/ERK signaling, indicated by pERK and pELK-1 expression (Fig. 5d). The discrepancy might reflect the cell type- and stimulus-specific requirement of ERK for GLI1 activity and needs further investigation. Nevertheless, our results suggest that MEKK2/3 are required for FGF2-induced inhibition on GLI1 activity using genetically engineered Daoy cells.

The role of MEKK2 and MEKK3 in cancer has recently been explored [30–32]. MEKK2 and MEKK3 have been found to be overexpressed or amplified in several types of cancer [33, 34] and knockdown of MEKK2 or MEKK3 inhibited cell proliferation in breast cancer cell lines [35]. In this study, however, we disclosed an inhibitory role of MEKK2/3 on GLI1 activity, which can suppress the cell growth of Daoy, a Hh pathway-dependent medulloblastoma cell line. These seemingly paradoxical outcomes of genes or pathways in different cancer types might stem from complex tissue interactions or incomplete understanding molecular mechanism. Indeed, we found a negative correlation between MEKK2/3 expression and Hh pathway activity in clinical medulloblastoma datasets. Genetic and pharmacologic studies in murine models will provide important information on the molecular and cellular basis of MEKK2/3 in Hh-dependent tumorigenesis. Nevertheless, the development of Hh pathway inhibitors targeting the factors downstream of SMO, such as GLI proteins, has emerged as a promising therapeutic strategy for the treatment of Hh-dependent tumors.

Taken together, our current study identifies GLI1 as a substrate for MEKK2/3, provides a mechanism for GLI1 inactivation and establishes a crosstalk between FGF2 and Hh pathways. The identification of MEKK2/3 as regulators of GLI1 activity in mammalian Hh signaling also provides new targets to modulate Hh activity. Many diseases result from uncontrolled GLI1 activity that is independent of canonical Hh signaling. In this regard, new modulators of GLI1 function, such as MEKK2/3, offer a unique opportunity to advance our understanding of Hh signaling and develop new therapies for diseases due to aberrant GLI1 activity.

## Materials and Methods

### Reagents and plasmids

Cyclopamine was obtained from Selleck Chemicals and used at 5 μM. Cycloheximide was from Sigma-Aldrich. Puromycin and G418 were from Sangon Biotech. ATP-γ-S and *p*-nitrobenzyl mesylate (PNBM) were purchased from Abcam. FGF2 was from Peprotech and used at 50 ng/ml. Plasmids expressing hGLI1, MEKK2, and MEKK3 were constructed into hemagglutinin (HA)-tagged or Flag-tagged pCDNA3 or pLVX lentiviral vector. Point mutation constructs were generated by site-directed mutagenesis PCR. All plasmids were verified by sequencing. pSpCas9(BB)-2A-Puro (PX459) was a gift from Feng Zhang (Addgene plasmid # 48139). sgRNA and shRNA target sequences are in the Supplementary table 2. The rabbit polyclonal antibody against the phosphorylation of GLI1-S204 was produced using the synthetic phosphorylated peptides GDMSSPN(pSer)TGIQD-Cys as antigen and purified on a phosphopeptide column.

### Cell culture, transfection, and lentiviral transduction

NIH3T3, HEK293T, Daoy, and D341 Med cells were cultured in Dulbecco’s modified Eagle’s medium (DMEM) containing 10% fetal bovine serum (FBS, GIBCO) and 1% penicillin/streptomycin at 37 °C with 5% CO_2_ and tested for mycoplasma contamination. Cell viability was assayed using Cell Counting Kit-8 (Biotool, Shanghai) according to the manufacturer’s instructions.

### Quantitative reverse transcription-PCR

Total RNA purified from cell cultures was used for cDNA synthesis and amplification by real-time PCR according to the manufacturer’s instructions (SYBR Green, TaKaRa). The primer information is provided Supplementary table 2.

### Luciferase assay

For luciferase assay, cells were seeded in 12-well plate and transfected with the reporter, CMV-β-gal, and indicated plasmids. Thirty-six hours after transfection, cells were lysed and luciferase activity was measured using the Luciferase Assay System (Promega) following the manufacturer’s instructions. All luciferase activities were normalized to β-galactosidase activity. For Shh CM, ShhN plasmid (Addgene #37680) was transfected into HEK293T for the production of Shh CM. For the Shh CM stimulation, cells grew to 100% confluence, and were serum-starved in DMEM with 0.5% FBS overnight, then treated with Shh CM for 12–16 h.

### Immunoprecipitation, immunoblotting, and immunofluorescence

Cells lysates were incubated with agarose-conjugated anti-HA or anti-Flag magnetic beads overnight at 4 °C. Beads were washed three times and the eluates were separated by SDS-PAGE and transferred to polyvinylidene fluoride membranes (EMD Millipore). Western blotting images were captured by ChemiScope5600 (Clinx, Shanghai) with ECL substrate. Western blot was measured using ImageJ to determine the relative intensities of protein bands, which were normalized using the internal loading control protein.

NIH3T3 or DAOY cells were seeded on round glass coverslip in 24-well plates. After transfection or transduction, cells were fixed and proceed to regular immunofluorescence.

The following antibodies were used: GLI1(C68H3) rabbit mAb (CST, 3538); MEKK2 rabbit mAb (Abcam, ab33918); MEKK3 (Proteintech, 21072-1-AP); Sufu(C81H7) rabbit mAb (CST, 2522); Tubulin rabbit mAb (Abcam, ab6046); GAPDH(6C5) (Abcam, ab8245); p-ERK1/2 rabbit mAb (CST, 4370); ERK1/2 (137F5) rabbit mAb (CST, 4695); Histone H3 (D1H2) rabbit mAb (CST, 4499); Thiophosphate ester rabbit mAb (Abcam, ab92570); Flag M2 mouse mAb (Sigma, F3165); HA (clone 3F10) rat mAb (Roche, 11815016001); p-ELK1 (Santa Cruz, SC-8406); and anti-phospho-MEKK2/3 antibody [15] (a gift from Bing Su).

### In vitro kinase assay

Flag-tagged MEKK2 or MEKK3 and GLI1-HA proteins were obtained using TNT Quick Coupled Transcription/Translation Systems (Promega) or immunoprecipited from transfected HEK293T cells. Cells were lysed in lysis buffer and the lysates were incubated with anti-Flag magnetic beads overnight at 4 °C. Beads were washed by lysis buffer for three times, then washed with wash buffer (40 mM Hepes and 200 mM NaCl) once and with kinase buffer (30 mM Hepes, 50 mM KAC, and 5 mM MgCl2) once. Immunoprecipited kinases and GLI1 with beads were mixed in the presence of ATP-γ-S (500 μM). Reaction mixtures were shaken gently at 30 °C for 1 h. A volume of 1 μl 0.5 M EDTA (final concentration 20 mM, pH 8.0) was added to terminate the reaction at 30 °C for 5 min. PNBM (2.5 mM) was added at 25 °C for 40 min to form a thiophosphate ester side chain. Kinase activity was analyzed by immunoblotting with anti-Thiophosphate ester antibody.

### DNA pull-down assay

Biotinylated 3xGliBS-oligos were synthetized by Thermo Fisher Scientific. Biotin-3xGliBS-oligos and reverse oligos were annealed. Biotin-GBS oligo sequence: forward primer, GACAAGCAGGGAACACCCAAGTAGAAGCTCCGACAAGCAGGGAACACCCAAGTAGAAGCT and reverse primer: AGCTTCTACTTGGGTGTTCCCTGCTTGTCGGAGCTTCTACTTGGGTGTTCCCTGCTTGTC.

HEK293T cells were transfected with indicated plasmids. Thirty-six hours after transfection, cells were collected in DNA-binding buffer (10 mM Hepes (pH 7.5), 150 mM NaCl, 1 mM MgCl_2_, 0.5 mM EDTA, 0.1% NP-40, 10% glycerol, 1 mM dithiothreitol, and 1 mM phenylmethylsulfonyl fluoride). Cell lysates were incubated with 50 pmol biotininylated double-strand oligos in a 500 μl volume at 37 °C for 30 min. DNA-bound GLI1 proteins were collected with Streptavidin beads (Thermo Fisher Scientific) at 4 °C for 1 h, then washed thoroughly with DNA-binding buffer, and boiled in 1× SDS-loading buffer for western blot analysis.

### Zebrafish experiments

Wild-type zebrafish embryos were injected with 100 pg RNA encoding human MEKK3 at one-cell stage. In situ hybridizations for *ptch1* were performed with digoxin-labeled RNA probe using standard protocol. Wild-type zebrafish embryos were coinjected with Cas9 mRNA and gRNAs for *mekk2* and *mekk*3 at one-cell stage. Embryos were collected for quantitative reverse transcription (RT)-PCR analysis at 36 hpf. DKO embryos for *mekk2* and *mekk*3 were genotyped by RT-PCR for the expression of *mekk2* and *mekk*3.

### Xenograft tumorigenesis model

Nude mice (nu/nu, male 6–8 weeks old) were randomly grouped and injected subcutaneously with 5 × 10^6^ Daoy cells. Tumor volume was measured every 5 days using the formula (*L* × *W*^2^)/2, where *L* is the longest diameter and *W* is the shortest diameter. The animal study protocols were approved by the Zhejiang University Animal Care and Use Committee.

### Patient data

Gene expression data for this study are accessible through public available GEO Series accession numbers GSE41842 and GSE37418.

### Statistics

Statistical analyses were performed with a two-tailed, unpaired Student’s *t*-test. When multiple comparisons were performed, one-way or two-way analyses of variance with Bonferroni-corrected Student’s *t*-tests as post tests were performed. A *P* value <0.05 was considered significant. *P < 0.05, **P < 0.01, ***P < 0.001. Except where otherwise indicated, experiments were repeated three times. Quantitative data were presented as mean ± s.d. from a representative of at least three independent experiments. All images shown were representative.

## Electronic supplementary material

Supplementary Figure 1-6

Supplemtary table 1

Supplemtary table 2

## References

[1] Briscoe J, Therond PP (2013). The mechanisms of Hedgehog signalling and its roles in development and disease. Nat Rev Mol Cell Biol.

[2] Jiang J, Hui CC (2008). Hedgehog signaling in development and cancer. Dev Cell.

[3] Wilson CW, Chuang PT (2010). Mechanism and evolution of cytosolic Hedgehog signal transduction. Development.

[4] Petrova R, Joyner AL (2014). Roles for Hedgehog signaling in adult organ homeostasis and repair. Development.

[5] Barakat MT, Humke EW, Scott MP (2010). Learning from Jekyll to control Hyde: Hedgehog signaling in development and cancer. Trends Mol Med.

[6] Scales SJ, de Sauvage FJ (2009). Mechanisms of Hedgehog pathway activation in cancer and implications for therapy. Trends Pharmacol Sci.

[7] Hui CC, Angers S (2011). Gli proteins in development and disease. Annu Rev Cell Dev Biol.

[8] Wang B, Fallon JF, Beachy PA (2000). Hedgehog-regulated processing of Gli3 produces an anterior/posterior repressor gradient in the developing vertebrate limb. Cell.

[9] Bai CB, Stephen D, Joyner AL (2004). All mouse ventral spinal cord patterning by hedgehog is Gli dependent and involves an activator function of Gli3. Dev Cell.

[10] Infante P, Alfonsi R, Botta B, Mori M, Di Marcotullio L (2015). Targeting GLI factors to inhibit the Hedgehog pathway. Trends Pharmacol Sci.

[11] Aberger F, Ruiz IAA (2014). Context-dependent signal integration by the GLI code: the oncogenic load, pathways, modifiers and implications for cancer therapy. Semin Cell Dev Biol.

[12] Cuevas BD, Abell AN, Johnson GL (2007). Role of mitogen-activated protein kinase kinase kinases in signal integration. Oncogene.

[13] Sasaki H, Nishizaki Y, Hui C, Nakafuku M, Kondoh H (1999). Regulation of Gli2 and Gli3 activities by an amino-terminal repression domain: implication of Gli2 and Gli3 as primary mediators of Shh signaling. Development.

[14] Sasaki H, Hui C, Nakafuku M, Kondoh H (1997). A binding site for Gli proteins is essential for HNF-3beta floor plate enhancer activity in transgenics and can respond to Shh in vitro. Development.

[15] Zhang D, Facchinetti V, Wang X, Huang Q, Qin J, Su B (2006). Identification of MEKK2/3 serine phosphorylation site targeted by the Toll-like receptor and stress pathways. EMBO J.

[16] Mao J, Maye P, Kogerman P, Tejedor FJ, Toftgard R, Xie W (2002). Regulation of Gli1 transcriptional activity in the nucleus by Dyrk1. J Biol Chem.

[17] Karlstrom RO, Tyurina OV, Kawakami A, Nishioka N, Talbot WS, Sasaki H (2003). Genetic analysis of zebrafish gli1 and gli2 reveals divergent requirements for gli genes in vertebrate development. Development.

[18] Lewis KE, Currie PD, Roy S, Schauerte H, Haffter P, Ingham PW (1999). Control of muscle cell-type specification in the zebrafish embryo by Hedgehog signalling. Dev Biol.

[19] Neumann CJ, Grandel H, Gaffield W, Schulte-Merker S, Nusslein-Volhard C (1999). Transient establishment of anteroposterior polarity in the zebrafish pectoral fin bud in the absence of sonic hedgehog activity. Development.

[20] Ding Q, Fukami S, Meng X, Nishizaki Y, Zhang X, Sasaki H (1999). Mouse suppressor of fused is a negative regulator of sonic hedgehog signaling and alters the subcellular distribution of Gli1. Curr Biol.

[21] Kogerman P, Grimm T, Kogerman L, Krause D, Unden AB, Sandstedt B (1999). Mammalian suppressor-of-fused modulates nuclear-cytoplasmic shuttling of Gli-1. Nat Cell Biol.

[22] Murone M, Luoh SM, Stone D, Li W, Gurney A, Armanini M (2000). Gli regulation by the opposing activities of fused and suppressor of fused. Nat Cell Biol.

[23] Barnfield PC, Zhang X, Thanabalasingham V, Yoshida M, Hui CC (2005). Negative regulation of Gli1 and Gli2 activator function by Suppressor of fused through multiple mechanisms. Differentiation.

[24] Greenblatt MB, Shin DY, Oh H, Lee KY, Zhai B, Gygi SP (2016). MEKK2 mediates an alternative beta-catenin pathway that promotes bone formation. Proc Natl Acad Sci USA.

[25] Fogarty MP, Emmenegger BA, Grasfeder LL, Oliver TG, Wechsler-Reya RJ (2007). Fibroblast growth factor blocks Sonic hedgehog signaling in neuronal precursors and tumor cells. Proc Natl Acad Sci USA.

[26] Gotschel F, Berg D, Gruber W, Bender C, Eberl M, Friedel M (2013). Synergism between Hedgehog-GLI and EGFR signaling in Hedgehog-responsive human medulloblastoma cells induces downregulation of canonical Hedgehog-target genes and stabilized expression of GLI1. PLoS ONE.

[27] Lin C, Chen MH, Yao E, Song H, Gacayan R, Hui CC (2014). Differential regulation of Gli proteins by Sufu in the lung affects PDGF signaling and myofibroblast development. Dev Biol.

[28] Di Marcotullio L, Ferretti E, Greco A, De Smaele E, Po A, Sico MA (2006). Numb is a suppressor of Hedgehog signalling and targets Gli1 for Itch-dependent ubiquitination. Nat Cell Biol.

[29] Huntzicker EG, Estay IS, Zhen H, Lokteva LA, Jackson PK, Oro AE (2006). Dual degradation signals control Gli protein stability and tumor formation. Genes Dev.

[30] Mazur PK, Reynoird N, Khatri P, Jansen PW, Wilkinson AW, Liu S (2014). SMYD3 links lysine methylation of MAP3K2 to Ras-driven cancer. Nature.

[31] Zhang W, Kong G, Zhang J, Wang T, Ye L, Zhang X (2012). MicroRNA-520b inhibits growth of hepatoma cells by targeting MEKK2 and cyclin D1. PLoS ONE.

[32] Umapathy G, El Wakil A, Witek B, Chesler L, Danielson L, Deng X (2014). The kinase ALK stimulates the kinase ERK5 to promote the expression of the oncogene MYCN in neuroblastoma. Sci Signal.

[33] Jiang L, Huang M, Wang L, Fan X, Wang P, Wang D (2013). Overexpression of MEKK2 is associated with colorectal carcinogenesis. Oncol Lett.

[34] Hasan R, Sharma R, Saraya A, Chattopadhyay TK, DattaGupta S, Walfish PG (2014). Mitogen activated protein kinase kinase kinase 3 (MAP3K3/MEKK3) overexpression is an early event in esophageal tumorigenesis and is a predictor of poor disease prognosis. BMC Cancer.

[35] Fan Y, Ge N, Wang X, Sun W, Mao R, Bu W (2014). Amplification and over-expression of MAP3K3 gene in human breast cancer promotes formation and survival of breast cancer cells. J Pathol.

