# Supplementary material for: MEKK2 and MEKK3 suppress Hedgehog pathway-dependent medulloblastoma by inhibiting GLI1 function
Source: Oncogene. 2018 Apr 17;37(28):3864–78. doi: 10.1038/s41388-018-0249-5 (PMC6041257; doi:10.1038/s41388-018-0249-5)
Supplement: Supplementary file 1 — Supplementary Figure 1-6 [file 41388_2018_249_MOESM1_ESM.pdf]

Supplementary Figure S1

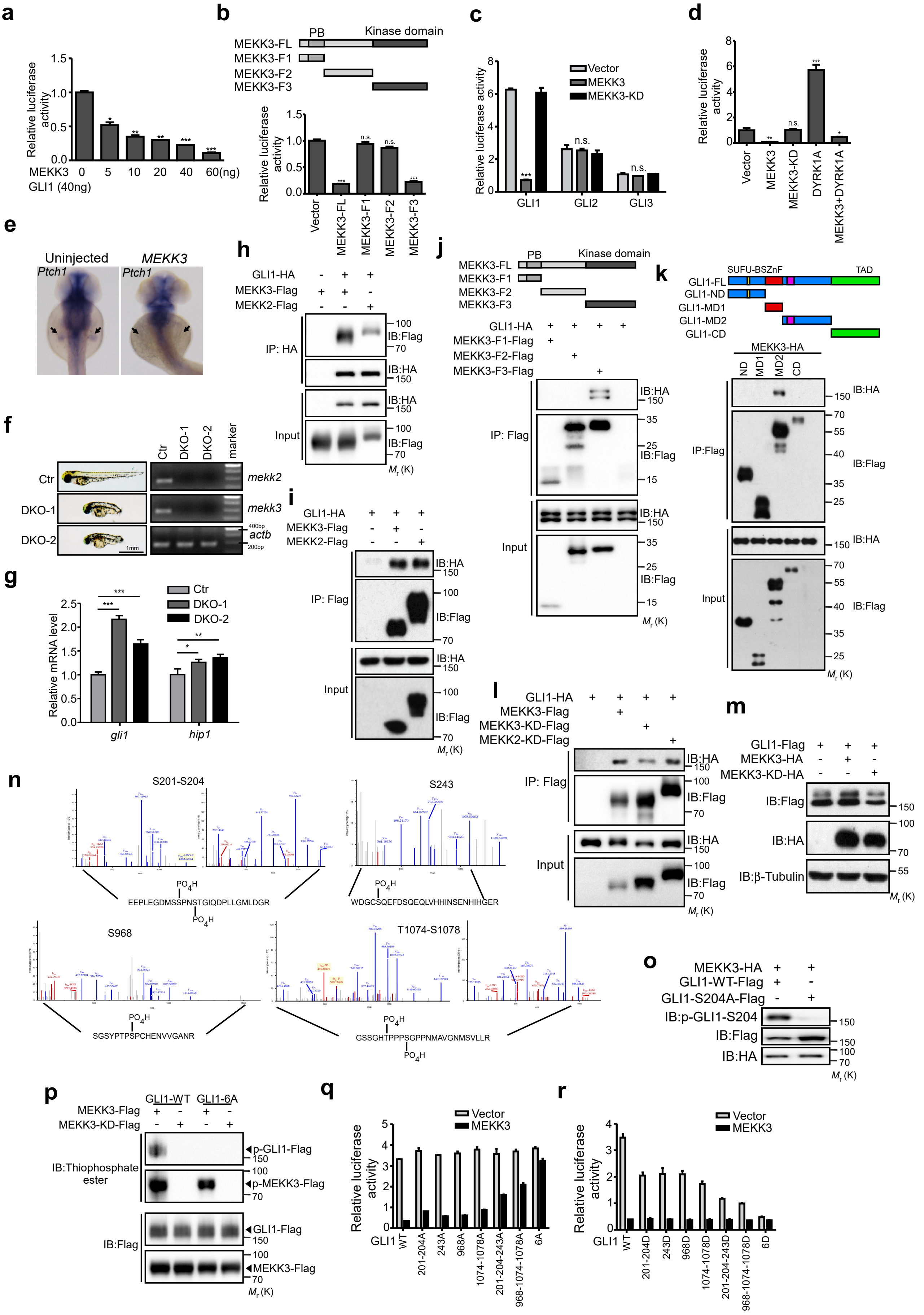

Supplementary Figure S1. MEKK2/3 specifically inhibit GLI1 transcriptional activity, associate with and phosphorylate GLI1

(a) MEKK3 inhibited GLI1 transcriptional activity in a dose-dependent manner. HEK293T cells were co-transfected with GliBS-luc reporter, GLI1 and MEKK3. Cell lysates were analyzed using a luciferase assay to measure GLI1 transcriptional activity. (b) The kinase domain of MEKK3 inhibited GLI1 transcriptional activity. A diagram shows the truncated mutants of MEKK3 (Upper panel). HEK293T cells were co-transfected with GliBS-luc reporter, GLI1, and MEKK3 truncated mutants. Cell lysates were analyzed by luciferase assay. (c) MEKK3 did not inhibit GLI2 and GLI3 transcriptional activity using GliBS-luc reporter assay in HEK293T cells. (d). MEKK3 inhibited DYRK1A induced Gli1 transcriptional activity. HEK293T cells were co-transfected with GliBS-luc reporter, GLI1 and indicated plasmids. Cell lysates were analyzed using a luciferase assay. (e) Whole-mount in situ hybridization of *ptch1* in uninjected zebrafish embryos and embryos injected with human *MEKK3* mRNA. Embryos were collected at 42 h post-fertilization (hpf). *ptch1* expression in the fin buds indicated by arrows was significantly reduced in embryos injected with *MEKK3* mRNA (n=35/47). (f) The gross appearance of the DKO zebrafish embryos for *mekk2* and *mekk3*. RT-PCR analysis revealed the lack of expression of *mekk2* and *mekk3* in DKO zebrafish embryos. (g) qPCR analysis of *gli1* and *hip1* expression in control and DKO zebrafish exbryos. (h) and (i) Co-immunoprecipitation assay revealed that GLI1 forms complexes with MEKK2 and MEKK3. Lysates from HEK293T cells transfected with indicated plasmids were immunoprecipitated and immunoblotted as indicated. (j) Kinase domain of MEKK3 associated with GLI1 in a co-immunoprecipitation assay using a series of MEKK3 deletion mutants in HEK293T cells. (k) A series of GLI1 deletion mutants were constructed as indicated and transfected into HEK293T cells together with MEKK3-HA. Cell lysates were immunoprecipitated with anti-Flag antibody and then subjected to western blot analysis using indicated antibodies. SUFU-BS, SUFU binding site; ZnF, zinc finger domain; NLS, nuclear localization signal; NES; nuclear export signal; TAD, transcriptional-activation domain. (l) GLI1 associated with kinase dead forms of

MEKK2 and MEKK3 in a co-immunoprecipitation assay in HEK293T cells. (m) MEKK3 induced a mobility shift of exogenous GLI1 in HEK293T cells. (n) Mass spectrometric analysis of GLI1 phosphorylation by MEKK3. HEK293T cells were co-transfected with GLI1-Flag and MEKK3-HA plasmids. The lysates were subjected to immunoprecipitation using anti Flag antibody, and GLI1 protein band was isolated and subjected to mass spectrometry. Extracted ion chromatograms (EICs) identified phosphorylated peptides at S201, S204, S243, S968, T1074 and S1078 in GLI1. (o) p-GLI1-S204 antibody recognized WT GLI1-Flag but not GLI1-S204A-Flag co-expressed with MEKK3 in HEK293T cells, indicating that this antibody is highly specific. (p) GLI1-6A mutant was resistant to phosphorylation by MEKK3 in an *in vitro* kinase assay. (q) and (r) GliBS-luc reporter assay evaluated the contribution of different phosphorylation sites to the GLI1 transcriptional activity in HEK293T cells. *P < 0.05, **P < 0.01 and ***P < 0.001 (two-tailed Student's t-test). All data were mean ± s.d. from representative of three independent experiments conducted in triplicate.

Supplementary Figure S2

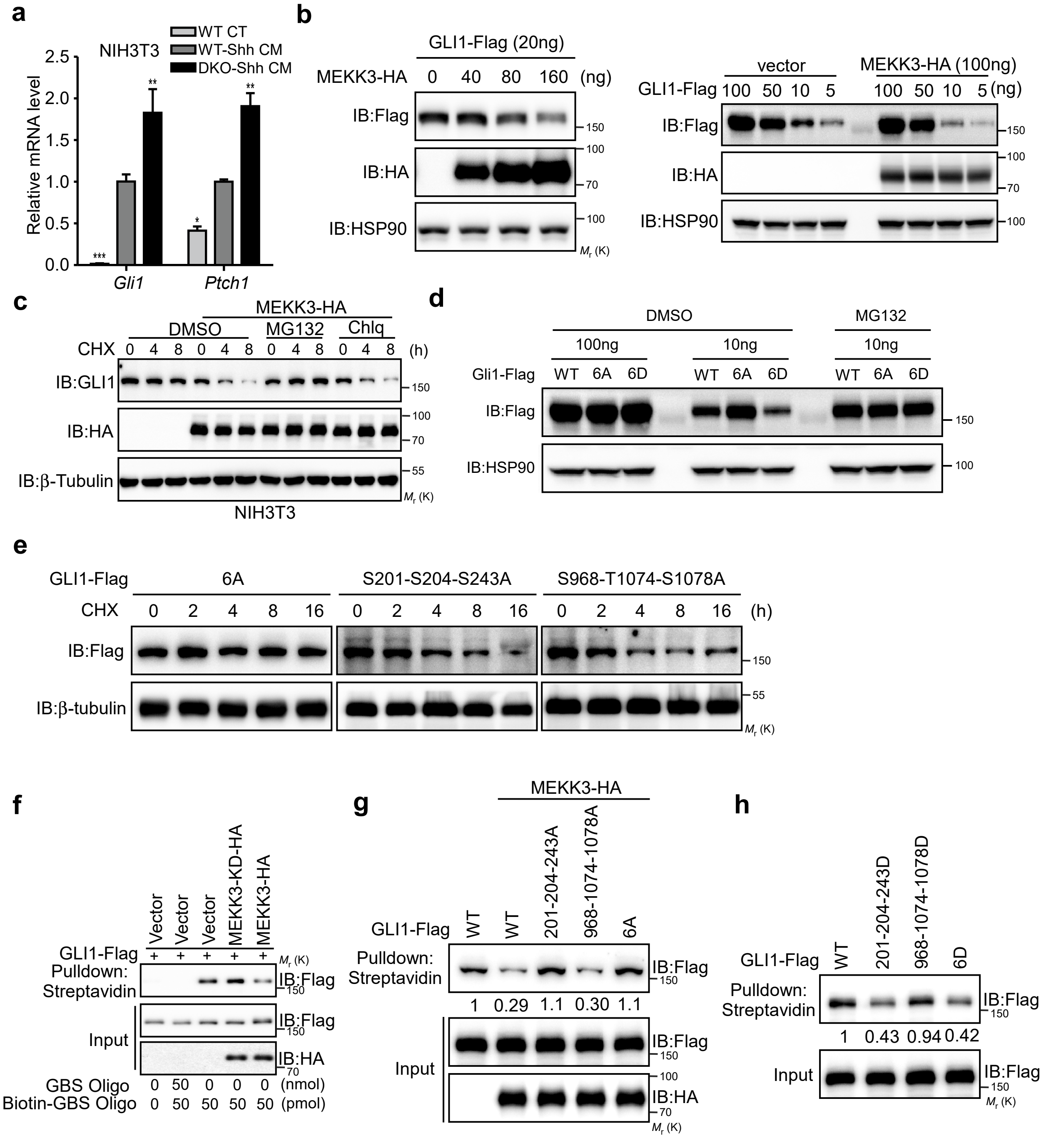

Supplementary Figure S2. **MEKK2/3 destabilize GLI1 protein and inhibit GLI1 DNA binding ability**

(a) Shh treatment induced higher expression of target genes in DKO NIH3T3 cells by qRT-PCR analysis. (b) Expression of MEKK3 promoted the degradation of exogenous GLI1 in HEK293T. HEK293T cells transfected with indicated amount plasmids and cell lysates were analyzed by western blot with the indicated antibodies. (c) MG132 (proteasome inhibitor) restored endogenous GLI1 protein levels in MEKK3 overexpressed NIH3T3 cells with the indicated antibodies. Chlq: Chloroquine (lysosomal inhibitor). (d) GLI1-6A was considerably stable, while GLI1-6D, phosphomimetic mutant, was unstable in HEK293T cells when lower amount of plasmid DNA was used for transfection. MG132 treatment restored the GLI1-6D protein level similar to GLI1-WT and GLI1-6A. HEK293T cells transfected with indicated amount plasmids and cell lysates were analyzed by western blot with the indicated antibodies. (e)HEK293T cells transfected with indicated plasmids were treated with cycloheximide (CHX, 20 μg/ml) for the indicated times, and cell lysates were analyzed by western blot with the indicated antibodies. (f) MEKK3, but not MEKK3-KD reduced DNA binding of GLI1 in a biotin-labeled DNA pull-down assay. Flag-GLI1 was efficiently precipitated by biotin-GliBS and non-labeled GliBS oligonucleotides abolished this binding, indicating the specificity of this assay. (g) DNA pull-down assay revealed that GLI1-S201/204/243A exhibited similar affinity with DNA as Gli1-6A in HEK293T cells. (h) DNA pull-down assay revealed that GLI1-S201/204/243D exhibited similar affinity with DNA as GLI1-6D in HEK293T cells.

Supplementary Figure S3

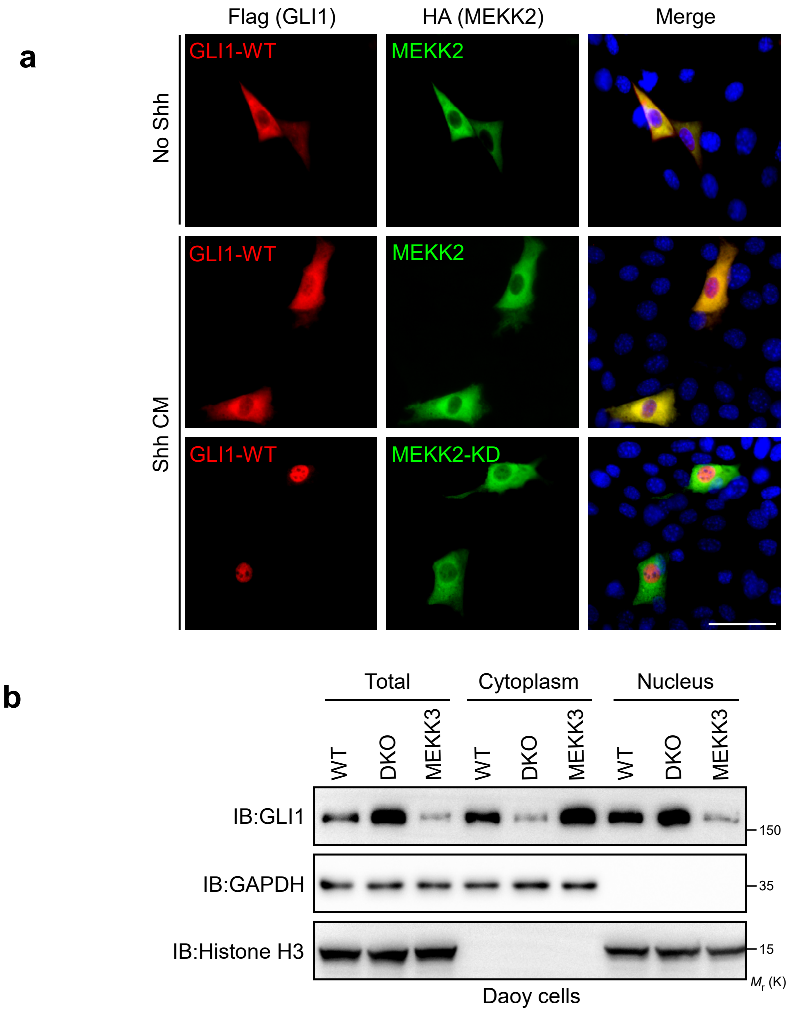

Supplementary Figure S3. **MEKK2/3 retain GLI1 in cytoplasm**

(a) Immunofluorescent analysis of the localization of GLI1 with MEKK2 in NIH3T3 cells treated with Shh CM. Scale bar = 20μm. (b) Western blot analysis for GLI1 protein levels in the nuclear and cytoplasmic fractions in DKO Daoy cells.

Supplementary Figure S4

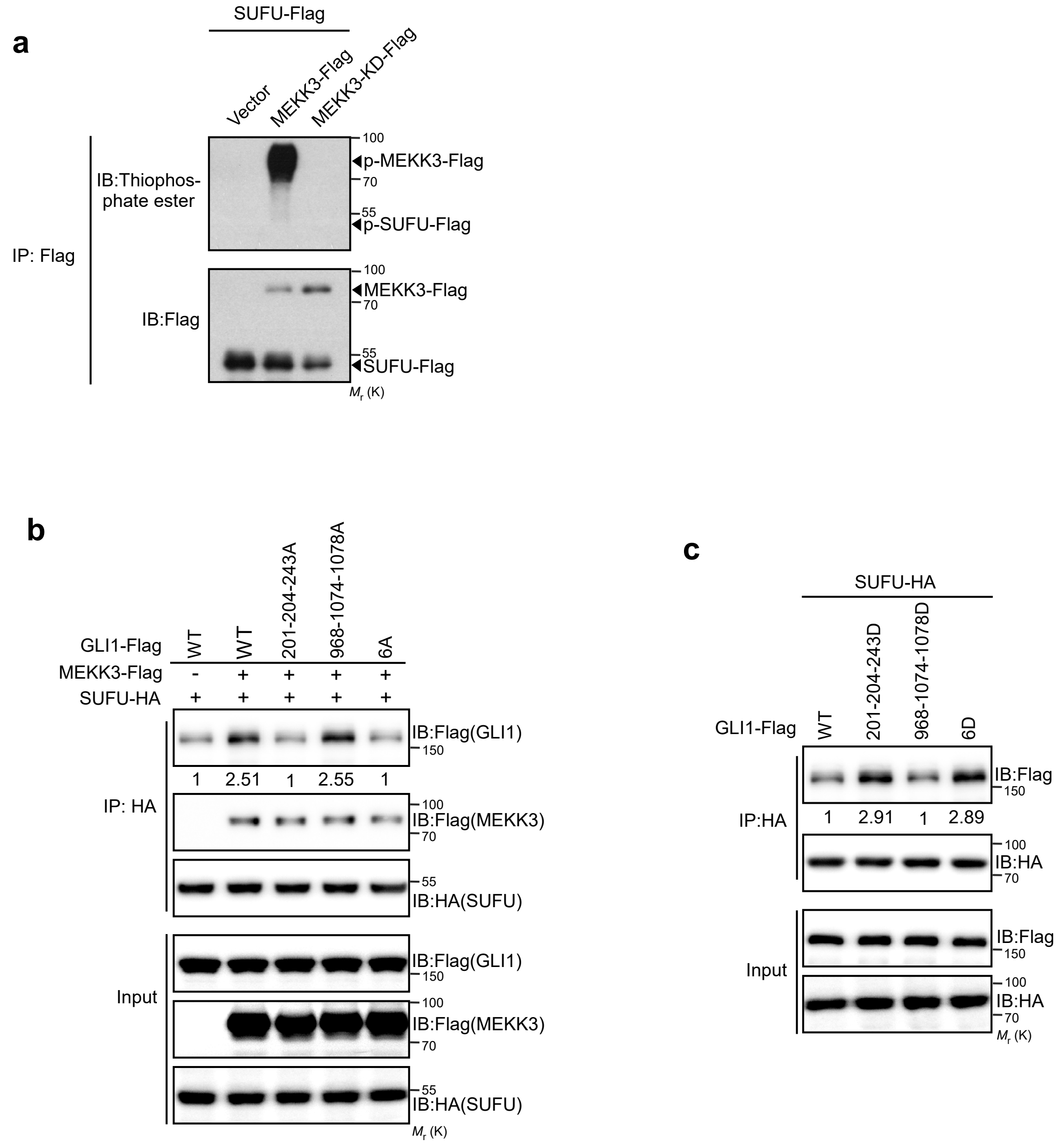

Supplementary Figure S4. **Phosphorylation of GLI1 by MEKK2/3 increases its binding with SUFU**

(a) SUFU was not a substrate for MEKK3 in an *in vitro* kinase assay. SUFU and MEKK3 kinase were immunoprecipitated from HEK293T cells individually, and an *in vitro* kinase assay was performed by mixing SUFU-Flag and MEKK3-Flag proteins in the presence of γ-S-ATP. No phosphorylation of SUFU was detected with thiophosphate ester antibody. Of note, this antibody identified the alkylated thiophosphorylation on MEKK3 but not MEKK3-KD. (b) Co-immunoprecipitation assay revealed that GLI1-S201/204/243A exhibited similar affinity with SUFU as GLI1-6A in HEK293T cells. (c) Co-immunoprecipitation assay revealed that GLI1-S201/204/243D exhibited similar affinity with SUFU as GLI1-6D in HEK293T cells.

Supplementary Figure S5

a

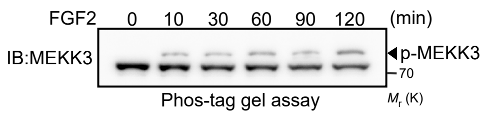

b

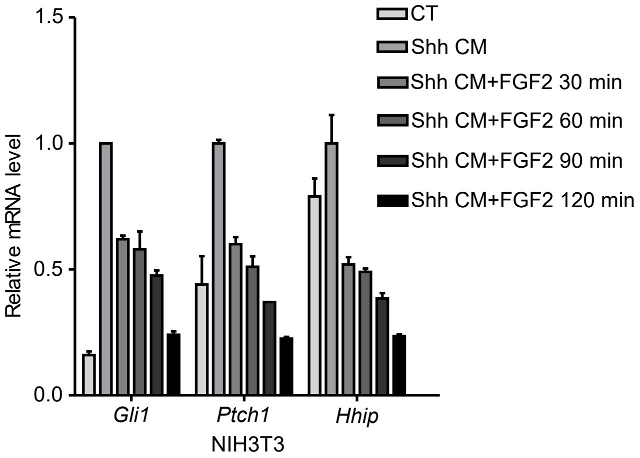

Supplementary Figure S5. **MEKK2 and MEKK3 are required for FGF2 mediated GLI1 transcriptional inhibition**

(a) NIH3T3 cells were treated with FGF2 for indicated time and cell lysates were run through phos-tag gel and analyzed by MEKK3 antibody. (b) Stimulation of Shh-primed Daoy cells with FGF2 induced downregulation of Hh/GLI target genes by qRT-PCR analysis. Daoy cells were pretreated with Shh CM for 9 h and then stimulated with FGF2 for indicated times. *$P < 0.05$, **$P < 0.01$ and ***$P < 0.001$ (two-tailed Student′s t-test). Quantitative data were presented as mean ± s.d. from a representative of at least three independent experiments.

Supplementary Figure S6

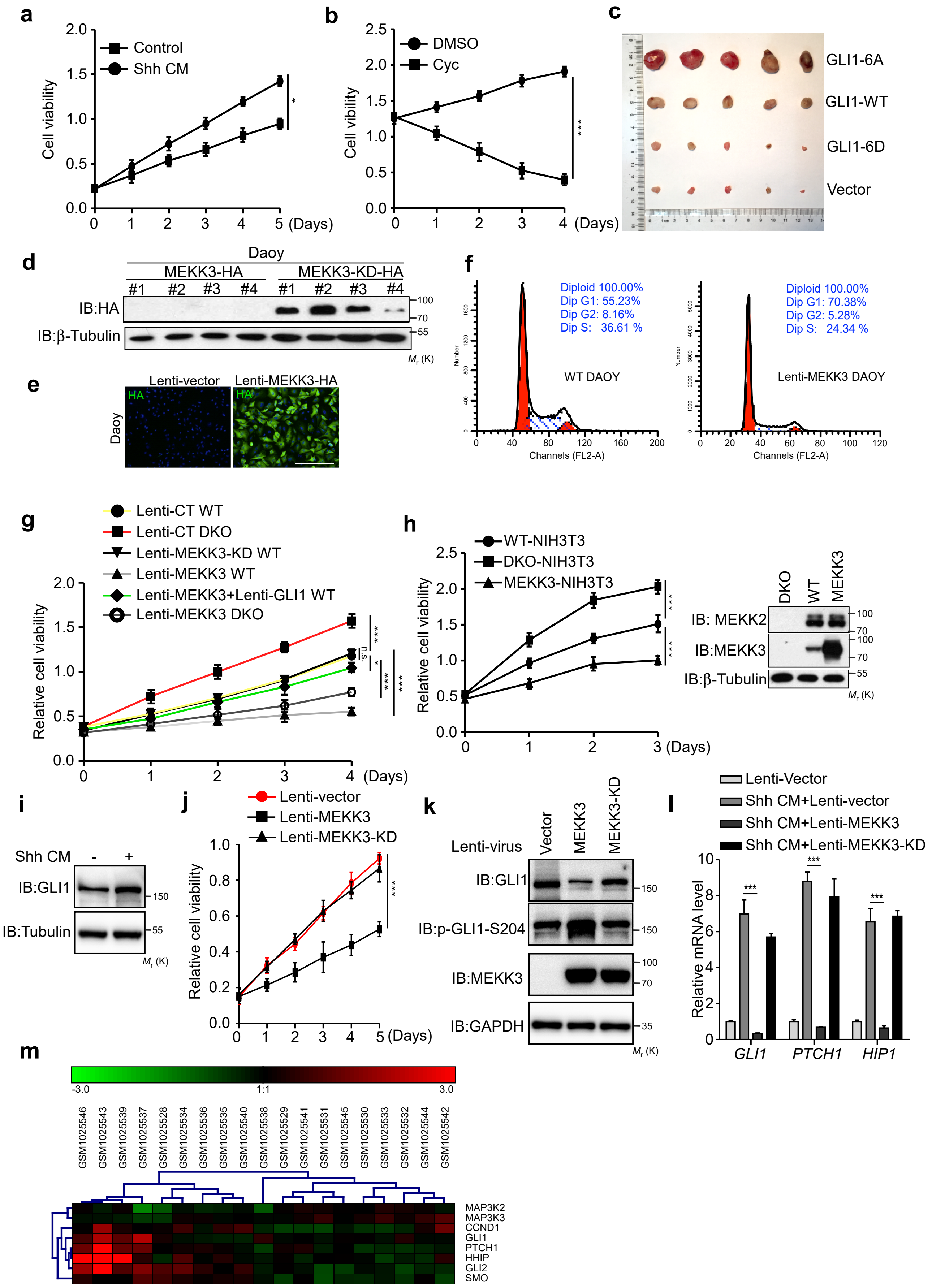

Supplementary Figure S6. **MEKK2 /3 inhibit Hh pathway dependent tumor cell proliferation**

(a) Shh promoted Daoy cell proliferation. Daoy cells were cultured in Shh CM for indicated times and subjected to cell proliferation assay using CCK8. (b) Daoy cells were cultured in Cyclopamine (5 μM) for indicated times and subjected to cell proliferation assay using CCK8. (c) The gross appearance of the tumors from nude mice injected with GLI1-WT, GLI1-6A and GLI1-6D Daoy cells was examined 20 days after transplantation. (d) Immunofluorescent analysis of transduction efficiency of lentivirus in Daoy cells. Daoy cells were fixed for immunofluorescent assay 2 days after lentivirus transduction with anti-HA antibody. (e) Western blot analysis of stable clones expressing MEKK3 and MEKK3-KD in Daoy cells. MEKK3 stable clones displayed undetectable MEKK3-HA expression. (f) Expression of MEKK3 resulted in G1 arrest in Daoy cells by flow cytometry analysis. (g) Effect of MEKK3 expression on Daoy cell proliferation. Daoy cells were transduced with indicated lentivirus and subjected to cell proliferation assay using CCK8. (h) Expression of MEKK3 inhibited cell proliferation in NIH3T3 cells. NIH3T3 cells were transduced with MEKK3 lentivirus and subjected to cell proliferation assay using CCK8. Expression of MEKK2 and MEKK3 was analyzed by western blot. (i) D341 Med cells treated with Shh CM were analyzed for the expression of GLI1 by western blot. (j) D341 Med cells were transduced with MEKK3 or MEKK3-KD lentivirus and subjected to cell proliferation assay using CCK8. (k) Western blot analysis for the expression of GLI1 in MEKK3 or MEKK3-KD lentivirus transduced D341 Med cells. (l) qPCR analysis for the expression of Hh pathway target genes in MEKK3 or MEKK3-KD lentivirus transduced D341 Med cells treated with Shh CM. (m) Heat map analysis reveals that expression of MEKK2/3 is negatively correlated with Hh pathway activity in GSE41842. $^*P < 0.05$, $^{**}P < 0.01$ and $^{***}P < 0.001$ (two-tailed Student′s t-test). Quantitative data were presented as mean ± s.d. from a representative of at least three independent experiments.
